# Effects of Multimodal Rheumatologic Complex Treatment in Childhood and Adolescence

**DOI:** 10.3390/children12040472

**Published:** 2025-04-07

**Authors:** Katharina Kreilinger, Regina Huehn, Jessica I. Hoell, Andreas Wienke, Katja Raberger

**Affiliations:** 1 Department of Pediatrics I, University Hospital, Martin-Luther-University Halle-Wittenberg, 06108 Halle (Saale), Germany; 2 Institute for Medical Epidemiology, Biometrics and Informatics, Martin-Luther-University Halle-Wittenberg, 06108 Halle (Saale), Germany

**Keywords:** multimodal complex treatment, childhood and adolescence, effects, juvenile idiopathic arthritis, somatoform disorder

## Abstract

Background: The aim of this study was to investigate the effects of multimodal rheumatologic complex treatment (MRCT) in childhood and adolescence. MRCT means a high-frequency treatment program of at least 11 h per week. Methods: MRCTs in children, carried out between May 2009 and May 2022 at the Department of Pediatrics of the University Hospital in Halle (Saale), were included in this study. The effects of the MRCT were evaluated based on inflammatory activity, functionality (using the Childhood Health Assessment Questionnaire (CHAQ)), subjective statements regarding pain intensity, state of health, and coping with the illness, as well as the objective determination of joint mobility. Data were analyzed retrospectively using *t*-tests to compare different groups and values before and after treatment. Results: During the study period, *N* = 133 MRCTs were conducted in *n* = 95 children. The most common diagnosis was juvenile idiopathic arthritis (83.2%). The c-reactive protein (CRP) fell from an average of 25.3 mg/L to 7.3 mg/L, and the erythrocyte sedimentation rate (ESR) fell from 29.5 mm in the first hour to 17.9 mm. Pain intensity was reduced from 5.4 to 4.0. The state of health and coping with the illness also improved. The disability index showed a moderate reduction from 0.92 to 0.81. Furthermore, an improvement in joint mobility was observed. Positive effects were also shown in patients with somatoform disorders. Conclusions: Due to the positive effects of MRCT on subjective well-being and physical health, the treatment program can be recommended for affected children, including patients with an additional diagnosed somatoform disorder.

## 1. Introduction

The prevalence of juvenile idiopathic arthritis (JIA) varies greatly depending on the region. In Germany, it is about 100 to 200 per 100,000 under-16 year olds [1,2,3,4]. According to the ILAR (International League of Associations for Rheumatology) criteria, JIA is diagnosed when the arthritis begins before the age of 16, lasts for at least six weeks, and other diseases presenting with similar symptoms have been excluded. Seven subtypes are currently distinguished: systemic arthritis, oligoarthritis (persistent or extended), seropositive polyarthritis, seronegative polyarthritis, psoriatic arthritis, enthesitis-associated arthritis, and unclassified arthritis. The latter is diagnosed when the arthritis cannot be assigned to any of the subtypes or has features of multiple subtypes [3,5]. In the case of children and adolescents with pronounced joint complaints due to a disease of the musculoskeletal system and/or a high subjective feeling of illness, multimodal rheumatologic complex treatment can be conducted in hospitals that fulfill the necessary structural characteristics. Patients stay in the hospital for at least seven days and receive therapy sessions for a minimum of eleven hours per week. While pediatric rheumatologists review or adjust the previous drug therapy, the children and adolescents receive physical and occupational therapy to improve joint function. The multimodal therapy concept is supplemented by cognitive behavioral therapy to support the young patients’ ability to cope with the illness. Furthermore, parents also receive information and psychological support regarding their child’s disease [5]. For adults, multimodal rheumatologic complex treatment has been included in the DRG (Diagnosis-related Groups) system since 2005 [6]. In recent years, positive effects have been shown for adult patients. A retrospective analysis of the effectiveness of multimodal complex treatment at the Rheumatism Center Bad Nauheim in adults with confirmed rheumatoid arthritis revealed a clear reduction in pain intensity and an improvement in functionality and disease activity [7]. Another study from the Rheumatism Center Rheinland-Pfalz also showed positive effects from multimodal treatment. Patients with rheumatoid arthritis, psoriatic arthritis, and ankylosing spondylitis were included in this study. An additional survey three months after treatment observed sustained positive effects related to pain intensity and duration of morning stiffness [8].

According to the current German guidelines from 2019, physiotherapy, occupational therapy, the provision of aids, and psychological support play an important role in the treatment of JIA, in addition to drug therapy. The use of thermotherapy, electrotherapy, lymphatic drainage, or massage can also be considered [9]. The American College of Rheumatology also recommends the use of physiotherapy and occupational therapy in the treatment of juvenile idiopathic arthritis, in addition to various groups of medication [10,11]. In 2006, multimodal rheumatologic complex treatment was also included in the DRG system for children and adolescents. So far, there are no publications on the effects of this treatment in children and adolescents, neither for Germany nor for other countries. To quantify the effects on physical health and subjective well-being, a retrospective analysis of complex treatments was performed in this study.

## 2. Materials and Methods

### 2.1. Study Design and Sample

For this study, data on conducted complex treatments between May 2009 and May 2022 at the Department of Pediatrics of the University Hospital in Halle (Saale) were retrospectively analyzed (pre–post design). The treatment can be offered to children and adolescents under the age of 18 with pronounced joint complaints due to musculoskeletal disorders and/or a high subjective feeling of illness.

The aim of the study was to investigate whether pain intensity, functionality, inflammatory activity, and joint mobility change during the period of treatment. During complex treatment, the inflammatory parameters ESR (erythrocyte sedimentation rate) after one hour and CRP (C-reactive protein) were determined at the beginning and the end of treatment. In addition, joint mobility was measured before and after treatment using the neutral-zero method, and functionality was determined using the CHAQ (Childhood Health Assessment Questionnaire) [12,13].

### 2.2. Components of the Multimodal Rheumatologic Complex Treatment

Since 2006, the components of the multimodal rheumatologic complex treatment in children and adolescents have been specified in the operation and procedure codes (OPSs), which is the official classification for coding treatment methods in Germany [14]. There must be a pediatric rheumatology specialist at the hospital, and at least three further types of therapy areas have to be offered. The following therapy areas are mentioned in the OPS: physiotherapy, physical therapy, occupational therapy, pain therapy, socio-pediatric care, and age-related cognitive behavioral therapy, including the teaching of disease management measures. All patients treated in the University Hospital Halle (Saale) have received physiotherapy, occupational therapy, pain therapy, and cognitive behavioral therapy. Only the density of the individual therapies varied from patient to patient depending on the symptoms. The duration of the therapies shall be at least 11 h per week. In total, the complex treatment lasts for at least seven days but can be extended to a maximum of three weeks. Furthermore, the operation and procedure code determines which values are to be measured by default. The CHAQ should be used to record functionality, while the visual analogue scale or the numerical rating scale should be used to assess pain intensity, depending on the age of the patient. In addition, the disease activity should be determined at the beginning and the end of complex treatment. According to the German guideline for the treatment of JIA, the determination of disease activity includes the determination of the acute phase parameters CRP or ESR, the assessment of the state of health, the number of joints with active arthritis, and the number of joints with restricted range of motion [9]. The minimum characteristics for the multimodal rheumatologic complex treatment also include weekly team meetings at which the treatment results are documented and further treatment goals are set [5].

### 2.3. Medication

Various drugs were used in the treatment of children and adolescents. The following groups of medications were considered for the evaluation: NSAIDs (nonsteroidal anti-inflammatory drugs), csDMARDs (conventional synthetic disease-modifying anti-rheumatic drugs), and bDMARDs (biological DMARDs), as well as glucocorticoids. Glucocorticoids can be used both as high-dose methylprednisolone pulses and as low-dose bridging agents until DMARDs take full therapeutic effect. There is also the option of intraarticular injection of glucocorticoids for the treatment of acute synovitis [15].

Not all drugs currently used for juvenile rheumatic disease have been available since 2009. Especially in the field of bDMARDs, more and more drugs have been authorized in recent years. While only adalimumab and etanercept were available at the beginning of complex treatment, there is now a large selection of bDMARDs that can be used depending on the subtype of juvenile idiopathic arthritis and individual tolerability [16,17].

### 2.4. Inflammatory Parameters

To assess inflammatory activity, CRP and ESR after one hour were determined before and after treatment. Only CRP values above 2.0 mg/L were relevant for the evaluation, as no further improvement was expected at lower values. For the ESR, values above 10 mm in the first hour were considered, as this is commonly used as a cut-off value for children [18]. Patients in whom an infection was detected during hospitalization were excluded from these calculations since an infection also influences the concentration of the inflammatory parameters and a change in these cannot be attributed entirely to the rheumatic disease. Both CRP and ESR are considered non-specific inflammatory parameters. There are many different causes for an increase in these values. In addition to rheumatic diseases, infectious inflammations also lead to an increase in these parameters [3]. To date, there is no specific laboratory parameter for assessing the inflammation activity of JIA.

### 2.5. Childhood Health Assessment Questionnaire and Additional Questions

The CHAQ is used to assess functionality in everyday life. It is divided into eight different domains of everyday life and contains a total of 30 questions. The children or their parents can choose one of four categories per question, depending on how severe the limitation in everyday life is. There is also the option to indicate that the child is still too young to be able to carry out the activity. There is always at least one task per domain that is relevant for children and adolescents of all ages, so a score can be calculated at the end. The highest score in each of the eight domains is used to calculate the disability index. It can take values between 0 and 3. The higher the value, the greater the restrictions in everyday life. A German version of the CHAQ was used for the children and adolescents in this study. The CHAQ is a reliable and valid instrument [12,19]. In this study, the standardized questionnaire was supplemented by several additional questions to detect the subjective complaints of the patients. These questions asked about pain intensity, perceived health status, and subjective coping with illness. The numerical rating scales range from 0 to 10. The higher the indicated value, the more severe the complaints are in each case. For the assessment of pain, this numerical rating scale is established in medicine. It is a reliable and valid method for assessing the intensity of pain [20,21].

### 2.6. Joint Status

At the beginning and the end of the complex treatment, the joint status of the patients was determined. The neutral-zero method was used to measure the range of motion for each joint. Using a defined neutral-zero position as a normal anatomical position, the mobility per movement axis can thus be specified for each joint. Three values result for each axis of movement, with the middle one describing the neutral-zero position. If this value deviates from the defined 0, there is a malposition of the joint in the corresponding movement axis [22]. Due to the lack of a standardized procedure for calculating the improvement in joint mobility, a separate method was developed in this study. To detect changes after the treatment, the deviations from the standard values were calculated. Values for the adult population were used as comparative values since values adapted to childhood were not documented [13].

In the evaluation, only those joints that clinically exhibited arthritis at the time of admission were considered. As soon as one of the five classic signs of inflammation (pain, redness, hyperthermia, swelling, or functional limitation) was present, the joint was classified as clinically conspicuous [23]. In addition, it was noted in which joints synovitis was detected based on the morphological imaging taken during the hospital stay. In arthrosonography, this is indicated by hypertrophy and/or increased vascularization of the synovium. Joint effusion alone was not sufficient for the diagnosis of synovitis. Using magnetic resonance imaging, synovitis can be diagnosed by increased contrast enhancement [3]. For evaluation, the affected joints were divided into two groups: first, those joints that showed synovitis on the morphological imaging, and second, the remaining joints with clinical abnormalities but without confirmed synovitis. For further analysis, all affected joints of patients with an additional documented somatoform disorder (ICD-10, Chapter V, Mental and Behavioral Disorders) were included. In this case, no distinction was made between joints with clinical arthritis or synovitis confirmed by morphological imaging.

In general, the following joints were measured using the neutral-zero method: shoulder, elbow, wrist, hip, knee, upper and lower ankle, and cervical spine. Since there have been no publications on joint status to compare values before and after treatment, a proprietary method was developed. All directions of motion were combined for each affected joint. The individual deviations from the standard values of the neutral-zero method were added per joint and the calculated mean values were compared before and after treatment.

### 2.7. Body Mass Index

The height and weight of the children and adolescents were used to calculate the body mass index (BMI). Using the standard values of the Robert Koch Institute for the corresponding age group, the children and adolescents were classified into five categories. Above the 97th percentile, patients are considered severely overweight; between the 90th and 97th percentiles, patients are considered overweight; between the 3rd and 10th percentiles, patients are considered underweight; and below the 3rd percentile, patients are considered severely underweight. Children whose age-specific BMI lies between the 10th and 90th percentiles are of normal weight [24].

### 2.8. Statistical Analysis

The analysis of the patient-specific data was performed using descriptive statistics. *T*-tests were used to calculate the effects of complex treatment. For sensitivity analyses, the calculations were also conducted using non-parametric tests. In addition, correlations were determined to analyze the influence of the duration of the disease on the patient’s complaints. For this purpose, the Pearson correlation coefficient was determined. The patient-related data refer to the number of patients. Only the data of the first treatment per patient were included in these statistics. In the presentation of treatment effects, the unit of observation is the totality of complex treatments, which, in some cases, includes several treatments for the same patient. For clarity purposes, n was used for the observation unit of patients and N for the observation unit of complex treatments. Finally, the evaluation of joint mobility refers to the number of included joints. All analyses were performed with the statistical program SPSS, version 27. For means, the 95% confidence intervals were reported.

## 3. Results

### 3.1. Patients

For this study, data from a total of 133 complex treatments (N = 133) conducted between May 2009 and May 2022 at the Department of Pediatrics of the University Hospital in Halle (Saale) were retrospectively analyzed. Every complex treatment carried out in the mentioned period was included in the analysis. Partial exclusions were made for some calculations due to missing data.

A total of 95 patients (*n* = 95) participated in one or more complex treatments in the above-mentioned period. The majority of the patients were girls (74.7%, *n* = 71). The mean age was 12.3 years (95% CI: 11.4; 13.3). The youngest child was 1.7 years old, and the oldest patient was 17.9 years old.

In 88.4% (*n* = 84) of the patients, a rheumatic diagnosis could be made prior to or during the complex treatment. In 83.2% (*n* = 79) of the patients, a subtype of juvenile idiopathic arthritis was present. This is by far the most frequent diagnosis of the patients in the multimodal rheumatologic complex treatment. In 11.6% (*n* = 11) of the children and adolescents, no clear diagnosis in the sense of a rheumatological disease could be made; only musculoskeletal or joint pain was documented. Appendix A shows the main diagnoses of first-time patients.

On average, the duration of the disease, meaning the time from diagnosis to the first participation in the complex treatment, was 16.3 months (95% CI: 10.9; 21.7). For the 11 pain patients without an exact diagnosis, the onset of pain was used as the time of diagosis. The proportion of first diagnoses during complex treatment was 30.5%.

For a more detailed description of the patients, comorbidities were also recorded. In order to avoid duplication, only patients participating in complex treatment for the first time were considered (*n* = 95). Nearly one-fifth of the first-time patients (18.9%, *n* = 18) were diagnosed with a somatoform disorder. Almost one in ten of the children and adolescents (9.5%, *n* = 9) suffered from bronchial asthma, 5.3% (*n* = 5) from atopic dermatitis, and 10.5% (*n* = 10) of the patients had hypothyroidism, which was treated with L-thyroxine. Two of these patients had a confirmed diagnosis of Hashimoto’s thyroiditis. In addition, two patients suffered from DiGeorge syndrome, which also increases the risk of autoimmune diseases [25]. Appendix B provides an overview of the comorbidities of the 95 patients.

As described in Section 2.7, the children and adolescents were classified into one of five BMI categories. In 76 first-time patients, a BMI could be calculated; in the other cases height or weight were not documented. On average, the BMI was 20.8 (95% CI: 19.4; 22.1). More than half of the children (60.5%, *n* = 46) were of a normal weight. In total, 15.8% of the patients were underweight or severely underweight, while 23.7% of the children were classified as overweight or severely overweight. Appendix C displays the exact distribution of the BMI categories.

### 3.2. Complex Treatment

The following calculations refer to the entirety of the complex treatments. A total of *N* = 133 multimodal rheumatologic complex treatments were performed in the above-mentioned period. The mean duration of treatment, including admission and discharge days, was 16.2 days (95% CI: 15.5;16.9).

#### 3.2.1. Drug Therapy

Different drugs are available for the treatment of juvenile idiopathic arthritis. Table 1 shows which medication groups were administered at the time of admission and discharge.

In 46.6% of cases, pediatric rheumatologists decided to change the patient’s baseline therapy. This included discontinuing or restarting one or more DMARDs. Table 2 shows which DMARDs were used at the beginning and the end of the complex treatment.

In addition, in 29.3% of the complex treatments, high-dose methylprednisolone pulses were administered by infusion over several hours on three consecutive days to further reduce inflammatory activity.

#### 3.2.2. Inflammatory Activity

In total, the CRP value at the beginning of treatment was above 2.0 mg/L in 52 complex treatments. In 39 cases, a CRP value was also documented at the end of the hospitalization, although 5 complex treatments were additionally excluded from the evaluation because the children concerned had an infection. Finally, *N* = 34 complex treatments were included in the evaluation. The children who were excluded from this calculation also showed inconspicuous values after complex treatment. The mean value for C-reactive protein for the 34 complex treatments mentioned above was 25.3 mg/L (95% CI: 16.3; 34.2) at the beginning of treatment and only 7.3 mg/L (95% CI: 4.2; 10.3) at discharge. A strong difference between the mean values before and after the complex treatment could be shown (*p* < 0.001).

The ESR is above 10 mm in one hour in 62 complex treatments at the beginning of treatment. After excluding four children with infections, the calculation could be performed for N = 40 complex treatments. In seven cases, the ESR values were increased at the end of treatment without a documented infection, although the values at the beginning of treatment, if present, were within the norm and thus not included in the calculation. On admission, the ESR for the mentioned 40 complex treatments averaged 29,5 mm (95% CI: 24.0; 25.0) in the first hour, while the average at discharge was 17.9 mm (95% CI: 14.1; 21.7) in the first hour. Once again, it was possible to show a difference between the inflammation values before and after the complex treatment (*p* < 0.001).

#### 3.2.3. Functionality in Daily Life

The disability index, which was measured using the CHAQ, could be determined for *N* = 55 treatments both at the beginning and at the end, as complete data were available. A moderate difference could be observed between the disability index before (M = 0.92; 95% CI: 0.75; 1.09) and after (M = 0.81; 95% CI: 0.64; 0.98) treatment (*p* = 0.048). In 52 cases, the questions on pain intensity and perceived health status were answered before and after complex treatment. Scale scores on subjective coping with illness were available in 51 cases before and after treatment. Pain intensity decreased from an average of 5.4 (95% CI: 4.7; 6.0) at admission to an average of 4.0 (95% CI: 3.3; 4.7) at discharge (*p* < 0.001). Perceived health status also showed an improvement. Before treatment, the average was 5.3 (95% CI: 4.6; 6.0), and, after treatment, it was only 3.6 (95% CI: 3.0; 4.2; *p* < 0.001). Subjective coping with illness also improved during the treatment. While the average was 4.3 (95% CI: 3.5; 5.0) at the beginning, it was only 3.0 (95% CI: 2.4; 3.6) at the end (*p* < 0.001).

The evaluation showed that patients with a somatoform disorder reported higher values of pain intensity at the beginning of treatment (*N* = 19; M = 7.2) compared to patients (*N* = 74; M = 4.9) without a documented somatoform disorder (*p* < 0.001). Also, the children and adolescents with somatoform disorder reported stronger pain (*N* = 12; M = 6.5) after the complex treatment than the other patients (*N* = 49; M = 3.2) (*p* < 0.001). Furthermore, there was no difference between pain intensity before (M = 7.2; 95% CI: 6.5; 7.9) and after complex treatment (M = 6.5; CI: 5.5; 7.5) in the children with somatoform disorders (*N* = 11; *p* = 0.236). However, the averages for health status differed strongly for patients with the somatoform disorder before (M = 7.5; 95% CI: 6.7; 8.2) and after (M = 5.7; 95% CI: 4.3; 7.0) complex treatment (*N* = 11; *p* = 0.027). The same is due to the subjective coping with illness. There was also a strong difference between the values at admission (M = 6.5; 95% CI: 4.5; 8.4) and at discharge (M = 4.2; 95% CI: 2.4; 5.9) (*N* = 10; *p* = 0.002).

In the following, the correlations between the duration of the disease and the disability index such as the information on pain intensity, health status, and coping with illness at the beginning of complex treatment were analyzed. Appendix D shows the results of these calculations.

A longer duration of the disease correlates with a lower scale value for the perceived coping with illness. A higher disability index correlates with higher values for perceived pain intensity, health status, and coping with illness. The three additional questions of the CHAQ also correlate with each other in the same direction.

#### 3.2.4. Frequency of Complex Treatments

The following Table 3 presents the frequency of complex treatments conducted in Halle (Saale) from 2010 to 2021.

It can be seen that fewer patients have received multimodal rheumatologic complex treatment in recent years.

### 3.3. Joint Mobility

On average, 1.7 joints (95% CI: 1.24; 2.09) showed synovitis on morphological imaging. The most affected joints were the knee (51), the wrist (50), the upper ankle (33), and the elbow (23). Since the other joints were rarely affected, the evaluation was limited to the joints just mentioned. In 21.1% (28) of the complex treatments, at least one joint was therapeutically punctured by instilling a synthetic glucocorticoid to specifically suppress inflammatory activity in the joint. The range was from one to eight punctured joints.

Table 4, Table 5 and Table 6 display the mean values of the deviations from the norm for the most frequently affected joints. Table 4 shows the calculated values for the different joints with synovitis proven by morphological imaging. The number of affected joints refers to those joints for which the joint status was available both before and after treatment. The other joints were excluded due to missing data.

Table 4 shows a strong decrease in the deviation from the normal values in the wrist, the knee, and the upper ankle. There was no improvement in joint function in the elbow joint.

Table 5 shows the corresponding values for the joints in which no synovitis could be detected by morphological imaging although symptoms such as pain or restricted movement were present.

Comparing both Table 4 and Table 5, it is noticeable that the changes in joint mobility are quite similar in both groups. Here, too, the wrist, the knee, and the upper ankle show considerably less deviation from the norm after treatment. As in the previous calculation, the elbow joint shows no improvement in joint function.

To detect the influence of the complex treatment on joint mobility in children with somatoform disorder, their affected joints were evaluated separately, in addition (Table 6). For this purpose, all joints with symptoms were grouped together. A total of 29 complex treatments involved patients with somatoform disorder.

The elbow joint, the wrist, and the upper ankle did not show any differences in joint mobility after treatment. However, there was also an improvement in the function of the knee joint in patients with somatoform disorders.

## 4. Discussion

The aim of this study was to analyze the effects of multimodal rheumatologic complex treatment in children and adolescents on pain intensity, inflammatory activity, functionality in everyday life, and joint mobility. It was also investigated whether patients with an additionally diagnosed somatoform disorder also benefit from the complex treatment. The inflammation parameters CRP and ESR decreased, and the pain intensity was reduced. There was also an improvement in joint mobility. The disability index showed only a moderate reduction. Positive effects on subjective well-being and physical health were also achieved in patients with additionally diagnosed somatoform disorders. The results of the study are discussed in detail below.

The gender ratio of patients in complex treatment also reflects the ratio for children and adolescents suffering from juvenile idiopathic arthritis. Girls are more frequently affected by most of the subtypes than boys. Given the small number of patients, the ratio of subtypes is about what would be expected in a German population. While there is no patient with systemic arthritis in this study group, the incidence is also quite low at 4–7% of all JIA cases in Europe. Most patients in the complex treatment had RF-negative polyarticular arthritis or oligoarticular arthritis (persistent or extended). These are also the two most common subtypes in Germany [3]. In addition, the relatively high proportion of unclassified arthritis in the total number of JIA cases is striking. In some cases, it was not possible to assign a clear subtype of juvenile idiopathic arthritis to the patients based on the data. This could be due to the fact that the children and adolescents received the rheumatologic complex treatment at the beginning of their disease and the further course had to be awaited for the exact determination of a subtype. Currently, a new classification method for JIA patients is being developed, which distinguishes between only five different categories [26]. It may then be easier to assign patients to a specific subtype. Recently, two large datasets have been analyzed showing the prevalence of JIA in Germany from 2013 to 2019. One of the aspects recorded was that of the comorbidities of the patients. While atopic dermatitis is less common, there are considerably more children with bronchial asthma and hypothyroidism among the patients of the complex treatment compared to the dataset mentioned [1]. Looking at the distribution of BMI, it is noticeable that more than one-third of first-time patients are underweight or overweight. Overall, nearly one in four children are shown to be overweight or even severely overweight. In Germany, the KiGGS Wave 2 was conducted from 2014 to 2017 and revealed that 7.6% of children and adolescents between the ages of 3 and 17 were underweight or severely underweight. In the same age group, 15.4% of children were overweight [27]. It is therefore noticeable that the patients of the complex treatment are more often below and above the normal weight. Several studies have already investigated the effects of obesity on JIA. Young adults with JIA who have an increased BMI show higher disease activity and lower functionality and report greater pain [28]. There is evidence that overweight JIA patients achieve a poorer remission rate under drug therapy with csDMARDs and bDMARDs than patients of normal weight. One reason for the poorer response to drug therapy in overweight patients could be that white adipose tissue produces pro-inflammatory cytokines [29].

There is ongoing progress in the field of drug therapy for JIA. Today, a wide range of biological DMARDs are commonly prescribed in addition to conventional synthetic DMARDs [16,30]. The choice of drug depends on many different criteria, for example, the subtype and individual tolerability of medication. The conventional synthetic DMARD methotrexate is still prescribed very frequently, which is also evident in the present study [17]. However, it should be noted that in 2009, when the first complex treatments were conducted, considerably fewer biological DMARDs were available. Therefore, the proportion of biological DMARDs administered in the following study group is lower than would currently be expected.

The presentation of the frequency of complex treatments conducted at the University Hospital in Halle (Saale) per calendar year shows that in recent years, fewer multimodal rheumatologic complex treatments were conducted for children and adolescents. This could be due to the improved quality of life of children and adolescents with JIA, which is made possible by the development of new drugs.

The inflammation values CRP and ESR in the first hour showed a strong decrease during the complex treatment. However, without a control group, it is not possible to say with certainty whether the decrease was due to the treatment alone. Furthermore, the number of cases in these calculations is low. In seven cases, the ESR value was above the norm after complex treatment, whereas it was normal at the beginning. In addition to inflammation, there are various reasons for the increase in this value. In women in particular, elevated ESR values can be measured shortly before menstruation, when body temperature changes, and when taking hormonal contraceptives. Laboratory errors can also be a reason for the increased values [18].

The disability index showed only a slight change after inpatient treatment. It is possible that the effects in everyday life will only become noticeable after a longer period of observation at home. However, it can also be assumed that everyday activities such as helping in the household or getting out of a vehicle cannot be properly assessed in this setting. To avoid this problem, the CHAQ should also be completed at home following treatment. At the next standard follow-up visit, parents could bring the questionnaire with them for evaluation. Furthermore, when calculating the disability index, the patient’s aids are also considered by default. If the functionality is assessed with a low score despite the need for aids, the actual value for this domain of everyday life is increased to two [12,19]. In the present study, the aids were not considered in the evaluation since the aids differ in part between admission and discharge, and comparability would thus no longer be given.

Pain intensity, perceived health status, and subjective coping with illness showed a strong improvement at discharge. This clearly demonstrates the positive effects of the complex treatment regarding pain intensity and the psychological well-being of children and adolescents. However, a weakness of our study is that it was not documented who filled out the questionnaire. It could have been the patients themselves, the children in collaboration with their parents, or a parent alone. Unfortunately, there were no precise regulations as to who should complete the questionnaire, e.g., regarding the age of the patients. Therefore, the possibility that the questionnaires were filled in by different people before and after treatment cannot be excluded. If we look at the correlations between the duration of the disease and the information on pain intensity, health status, and coping with the illness at the beginning of treatment, it is noticeable that children who have had longer courses of disease appear to be better able to cope with their illness.

As described above, it is noticeable that patients with a somatoform disorder reported greater pain both before and after treatment than those children without a somatoform disorder. Furthermore, the affected patients only showed a moderate improvement in pain intensity despite the intensive treatment. Somatization and pain disorders were grouped together as somatoform disabilities according to ICD-10. The results regarding pain intensity are therefore not surprising since the diagnosis is made when there is no subjective improvement, but, at the same time, the objective findings do not show sufficient justification for this [31]. In 2014, an article explained that patients with JIA have an increased pain intensity. Both subjective and objective parameters were evaluated [32]. In recent work, evidence was found that there is an alteration within neurocircuitry structures in patients with JIA. These may be responsible for altered pain processing [33]. However, in the present work, it was shown that the perceived health status and subjective coping with illness improved in patients with somatoform disorders as a result of the complex treatment. Although the sample in the above-mentioned evaluations is quite small, there is at least a trend that the complex treatment pays off at a subjective level in these children. It is not surprising that patients with somatoform disorders benefit from rheumatologic complex treatment, as the therapy for these disorders is also multimodal and includes physiotherapy, occupational therapy, pain therapy, and cognitive behavioral therapy.

It has long been known that regular physical activity has positive effects on patients with rheumatoid arthritis. Pain intensity is reduced and the quality of life and functionality in everyday life improve [34,35]. For young patients with JIA, data are sparse. However, a systematic review recently suggested that children and adolescents with JIA also benefit from physical interventions [36]. In this study, a special method was developed to visualize the change in joint mobility after complex treatment, as there is no standardized method for this to date. This evaluation of joint status showed that joint mobility in the investigated joints at discharge deviated less from the norm than before treatment. The improvement in joint function was seen not only in joints with proven synovitis, but also in symptomatic joints in which no synovitis could be detected on morphological imaging. Furthermore, it could be observed that the joints of children with somatoform disorder also benefit from the intensive complex treatment. Once again, the joints showed reduced abnormalities at the end of treatment, although to a lesser extent than in children without somatoform disorders. One reason for the less marked improvement in joint function in children and adolescents with somatoform disorder could be that joint mobility was already less restricted before treatment than in joints with proven synovitis. It may be more difficult to achieve an additional improvement in mobility in these joints. However, these children also showed at least a minimal improvement in joint function. It can therefore be said that patients with such a disorder appear to benefit not only mentally but also physically from the complex treatment. Nevertheless, it should not go unmentioned that, up to now, there has been no consistent definition of when one can speak of an improvement in joint function at all. It is conceivable that the method developed in this study for comparing joint function at different time points could be used in the future as a standard for assessing changes in joint function over time.

The results of the evaluation clearly show how much pain patients benefit from the multimodal rheumatologic complex treatment. The inpatient setting has the great advantage that the therapy sessions can be carried out daily. In the outpatient setting, physiotherapy, occupational therapy, and cognitive behavioral therapy are often only possible once a week at most, as the children and their parents are heavily involved in school, work, and other daily activities. In addition, therapists are frequently unable to offer patients the required number of therapy sessions. It is a big problem that there are too few therapists who specialize in children and adolescents. It would be desirable for the young patients if this would improve in the future.

### Limitations

Due to the weakness of the retrospective study design, there was unfortunately no control group. Additional randomized controlled trials would be desirable in the future to be able to draw further conclusions about the effects of multimodal rheumatologic complex treatment.

A new method for assessing joint mobility in the pre–post design has been developed; however, this method has not been validated.

Another limitation is that it is not possible to determine which of the applied therapy areas contributes the most to the positive effects of multimodal rheumatologic complex treatment, as the retrospective data analysis meant that only the data that were available or specified by the OPS in the pre–post design could be analyzed. However, the other forms of therapy (cognitive behavioral therapy and occupational therapy), which were also carried out but for which no pre–post comparisons are available, could certainly have a positive effect on the components examined. Prospective studies and multivariable analyses are desirable, considering all therapy areas carried out.

## 5. Conclusions

The retrospective analysis of the data showed positive effects of multimodal rheumatologic treatment in childhood and adolescence. Inflammatory activity was reduced, pain intensity decreased, and subjective sensation, as well as joint mobility, improved. A special method was developed in this study to be able to visualize changes in joint function after complex treatment. Positive effects on physical health and subjective well-being are also observed by patients with a diagnosed somatoform disorder, so a recommendation for multimodal rheumatologic complex treatment can also be made for these children and adolescents. The disability index also showed an improvement after treatment, albeit only to a small extent. To increase the validity of the disability index measurement, the questionnaire could be completed in the patient’s home after discharge.

## Figures and Tables

**Table 1:** Medication before and after complex treatment.

Medication Group	At Admission in %	At Discharge in %
Administered medication	59.4 (*N* = 79)	92.5 (*N* = 123)
NSAIDs	50.4 (*N* = 67)	86.5 (*N* = 115)
csDMARDs	15.0 (*N* = 20)	46.6 (*N* = 62)
bDMARDs	5.3 (*N* = 7)	3.8 (*N* = 5)
csDMARDs and bDMARDs	13.5 (*N* = 18)	17.3 (*N* = 23)
Glucocorticoids	19.5 (*N* = 26)	45.9 (*N* = 61)

Since in some cases, several medications were administered, the values do not add up to 100 percent. NSAIDs: nonsteroidal anti-inflammatory drugs. csDMARDs: conventional synthetic disease-modifying anti-rheumatic drugs. bDMARDs: biological disease-modifying anti-rheumatic drugs.

**Table 2:** DMARDs before and after complex treatment.

Medication	At Admission in %	At Discharge in %
csDMARDs		
Methotrexate	24.1 (*N* = 32)	53.4 (*N* = 71)
Sulfasalazine	5.3 (*N* = 7)	12.8 (*N* = 17)
bDMARDs		
Etanercept	9.0 (*N* = 12)	11.3 (*N* = 15)
Adalimumab	6.0 (*N* = 8)	6.0 (*N* = 8)
Tocilizumab	1.5 (*N* = 2)	1.5 (*N* = 2)
Abatacept	0.8 (*N* = 1)	1.5 (*N* = 2)
Infliximab	0.8 (*N* = 1)	0.8 (*N* = 1)
Golimumab	0.8 (*N* = 1)	0.0 (*N* = 0)

DMARDs: disease-modifying anti-rheumatic drugs.

**Table 3:** Frequency of complex treatments from 2010 to 2021.

Year of Treatment	Number of Complex Treatments
2009 *	6
2010	12
2011	21
2012	17
2013	10
2014	8
2015	15
2016	10
2017	10
2018	6
2019	3
2020	5
2021	7
2022 *	3

* In 2009 and 2022, the observation period was less than 12 months, as the data were analyzed from May 2009 to May 2022 (*N* = 133).

**Table 4:** Joint mobility in joints with confirmed synovitis.

Joint	Number of Affected Joints	Main Value Before Treatment (in °)	Main Value After Treatment (in °)	*p*-Value	Number of Directions of Motion
Elbow	18	43.5	30.3	*p* = 0.066	2
Wrist	40	44.9	32.5	*p* < 0.001	2
Knee	50	13.2	7.0	*p* = 0.008	1
Upper ankle	32	29.8	24.8	*p* < 0.001	1

Elbow: flexion/extension and pronation/supination; wrist: dorsal flexion/palmar flexion and radial deviation/ulnar deviation; knee: flexion/extension; upper ankle: dorsal flexion/plantar flexion.

**Table 5:** Joint mobility in joints with symptoms but without confirmed synovitis.

Joint	Number of Affected Joints	Main Value Before Treatment (in °)	Main Value After Treatment (in °)	*p*-Value	Number of Directions of Motion
Elbow	21	43.7	36.7	*p* = 0.238	2
Wrist	58	28.4	20.3	*p* < 0.001	2
Knee	50	14.3	9.5	*p* < 0.001	1
Upper ankle	58	22.7	19.8	*p* = 0.001	1

Elbow: flexion/extension and pronation/supination; wrist: dorsal flexion/palmar flexion and radial deviation/ulnar deviation; knee: flexion/extension; upper ankle: dorsal flexion/plantar flexion.

**Table 6:** Joint mobility in affected joints in children with somatoform disorder.

Joint	Number of Affected Joints	Main Value Before Treatment (in °)	Main Value After Treatment (in °)	*p*-Value	Number of Directions of Motion
Elbow	9	24.2	21.1	*p* = 0.790	2
Wrist	25	25.1	19.2	*p* = 0.061	2
Knee	21	22.1	12.1	*p* = 0.027	1
Upper ankle	13	25.8	23.5	*p* = 0.139	1

Elbow: flexion/extension and pronation/supination; wrist: dorsal flexion/palmar flexion and radial deviation/ulnar deviation; knee: flexion/extension; upper ankle: dorsal flexion/plantar flexion.

## Data Availability

The datasets used and/or analyzed during the current study are available from the corresponding author upon reasonable request.

## Abbreviations

JIA	Juvenile idiopathic arthritis
ILAR	International League of Associations for Rheumatology
DRG	Diagnosis-related groups
ESR	Erythrocyte sedimentation rate
CRP	C-reactive protein
CHAQ	Childhood Health Assessment Questionnaire
OPS	Operation and procedure codes
BMI	Body mass index
RF	Rheumatoid factor
SLE	Systemic lupus erythematosus
CRMO	Chronic recurrent multifocal osteomyelitis
NSAID	Nonsteroidal anti-inflammatory drug
csDMARD	Conventional synthetic disease-modifying anti-rheumatic drug
bDMARD	Biological disease-modifying anti-rheumatic drug
N	Number of complex treatments
n	Number of patients

## Appendix A. Main Diagnoses of the Patients

**Diagnoses**	**Number of Patients (n)**	**Proportion of Patients (in %)**
**Persistent oligoarthritis**	15	15.8
**Extended oligoarthritis**	6	6.3
**RF-positive polyarthritis**	2	2.1
**RF-negative polyarthritis**	26	27.4
**Psoriatic arthritis**	2	2.1
**Enthesitis-associated arthritis**	13	13.7
**Unclassified arthritis**	15	15.8
**SLE**	1	1.1
**Behçet’s disease**	1	1.1
**Scleroderma**	1	1.1
**CRMO**	1	1.1
**Fibromyalgia**	1	1.1
**Arthralgia**	11	11.6
**Summary**	95	100
RF: rheumatoid factor. SLE: systemic lupus erythematosus. CRMO: chronic recurrent multifocal osteomyelitis.		

## Appendix B. Comorbidities of the Patients

**Comorbidities**	**Number of Patients (n)**	**Proportion of Patients (in %)**
**Somatoform disorder**	18	18.9
**Bronchial asthma**	9	9.5
**Atopic dermatitis**	5	5.3
**Hypothyroidism**	10	10.5
**DiGeorge syndrome**	2	2.1

## Appendix C. BMI Categories of the Patients

**BMI Categories**	**Number of Patients (n)**	**Proportion of Patients (in %)**
**Severely underweight**	8	10.5
**Underweight**	4	5.3
**Normal weight**	46	60.5
**Overweight**	12	15.8
**Severely overweight**	6	7.9
**Summary**	76	100
BMI: Body mass index.		

## Appendix D. Correlation of the Duration of the Disease with the Additional Questions of the CHAQ

		**Duration of the Disease**	**Disability Index Before Treatment**	**Perceived Pain Intensity Before Treatment**	**Perceived Health Status Before Treatment**	**Perceived Coping with Illness Before Treatment**
**Duration of the disease**	correlation	1	−0.031	−0.107	−0.118	−0.232
	*p*-value		0.765	0.307	0.261	0.026
	N	133	95	93	93	92
**Disability index before treatment**	correlation	−0.031	1	0.321	0.513	0.304
	*p*-value	0.765		0.002	0	0.003
	N	95	95	92	92	91
**Perceived pain intensity before treatment**	correlation	−0.107	0.321	1	0.596	0.66
	*p*-value	0.307	0.002		0	0
	N	93	92	93	93	92
**Perceived health status before treatment**	correlation	−0.118	0.513	0.596	1	0.593
	*p*-value	0.261	0	0		0
	N	93	92	93	93	92
**Perceived coping with illness before treatment**	correlation	−0.232	0.304	0.66	0.593	1
	*p*-value	0.026	0.003	0	0	
	N	92	91	92	92	92
CHAQ: Childhood Health Assessment Questionnaire.						
